# Nursing students’ experiences of a pedagogical transition from campus learning to distance learning using digital tools

**DOI:** 10.1186/s12912-021-00542-1

**Published:** 2021-01-19

**Authors:** Ulrica Langegård, Kiana Kiani, Susanne J. Nielsen, Per-Arne Svensson

**Affiliations:** 1 Institute of Health and Care Sciences, Sahlgrenska Academy at University of Gothenburg, Arvid Wallgrens backe, Box 457, 405 30 Göteborg, Sweden; 2 Department of Molecular and Clinical Medicine, Institute of Medicine, Sahlgrenska Academy at University of Gothenburg, Gothenburg, Sweden

**Keywords:** Teaching [MeSH], Education, Professional [MeSH], Distance learning, Digital tools, Quantitative method, Qualitative method, Social interaction [MeSH], Blended learning

## Abstract

**Background:**

The use of distance education using digital tools in higher education has increased over the last decade, particularly during the COVID-19 pandemic. Therefore, this study aimed to describe and evaluate nursing students’ experiences of the pedagogical transition from traditional campus based learning to distance learning using digital tools.

**Methods:**

The nursing course *Symptom and signs of illness* underwent a transition from campus based education to distance learning using digital tools because of the COVID-19 pandemic. This pedagogical transition in teaching was evaluated using both quantitative and qualitative data analysis. Focus group interviews (*n* = 9) were analysed using qualitative content analysis to explore students’ experiences of the pedagogical transition and to construct a web-based questionnaire. The questionnaire comprised 14 items, including two open-ended questions. The questionnaire was delivered to all course participants and responses were obtained from 96 of 132 students (73%). Questionnaire data were analyzed using descriptive statistics and comments from the open-ended questions were used as quotes to highlight the quantitative data.

**Results:**

The analysis of the focus group interviews extracted three main dimensions: *didactic aspects of digital teaching*, *study environment*, and *students’ own resources. Social interaction* was an overall theme included in all three dimensions. Data from the questionnaire showed that a majority of students preferred campus based education and experienced deterioration in all investigated dimensions after the pedagogical transition. However, approximately one-third of the students appeared to prefer distance learning using digital tools.

**Conclusions:**

The main finding was that the pedagogical transition to distance education reduced the possibility for students’ social interactions in their learning process. This negatively affected several aspects of their experience of distance learning using digital tools, such as reduced motivation. However, the heterogeneity in the responses suggested that a blended learning approach may offer pedagogical benefits while maintaining an advantageous level of social interaction.

**Supplementary Information:**

The online version contains supplementary material available at 10.1186/s12912-021-00542-1.

## Background

The use of distance learning in higher education institutions has expand globally [1]. Distance learning using digital tools can be defined as “the use of electronic technology to deliver, support and enhance both learning and teaching and involves communication between learners and teachers utilizing online content” [2]. Distance learning may facilitate a pedagogical transition from a teacher-centered approach in which lectures may results in a one-way communication, to a learner-centered approaches which involve the student’s interaction with their teachers and other students. Education via distance learning with digital tools can facilitate variability in learning situations and course content [3, 4].

In nursing education, campus based lectures are a major part of the learning activities [5]. A campus based lecture approach can manifest in a teaching culture and become pervasive within an organization or discipline, leading to a reluctance to adopt new and emerging practices and technologies [6]. Barriers to implementation and use of distance learning in nursing education may be related to teachers’ limited experience and knowledge in using digital tools when organizing learning activities. The implementation can also be affected by teachers’ fear that distance education may reduce or remove traditional campus based lectures [6].

The pedagogical transition from traditional to distance learning is a challenge for nursing education. Experiences from courses that included a combined pedagogical approach with both distance and campus based learning showed that students found campus based education valuable for their learning [7]. Compared with distance learning only, a blended learning approach including campus based learning and distance learning, may give students increased motivation in their learning process [8]. A literature review revealed that online learning in nursing education was as effective as traditional campus based learning [9]. In addition, previous studies reported contradictory or equivalent results regarding the benefits and hindrances of traditional campus based learning and distance learning using digital tools for nursing education [10, 11].

As a consequence of the COVID-19 outbreak during spring 2020, a number of universities worldwide were forced to rapidly change the pedagogical approach from traditional campus based learning to distance learning using digital tools. This change constituted a major challenge for both teachers and students and warrants extensive evaluation. Overall, more knowledge about students’ experiences when using different pedagogical approaches, including distance learning, is needed to improve didactic strategies in nursing education. Therefore, this study aimed to describe and evaluate nursing students’ experiences of pedagogical transition from traditional campus based learning to distance learning using digital tools.

## Methods

### Design

**Fig. 1:**
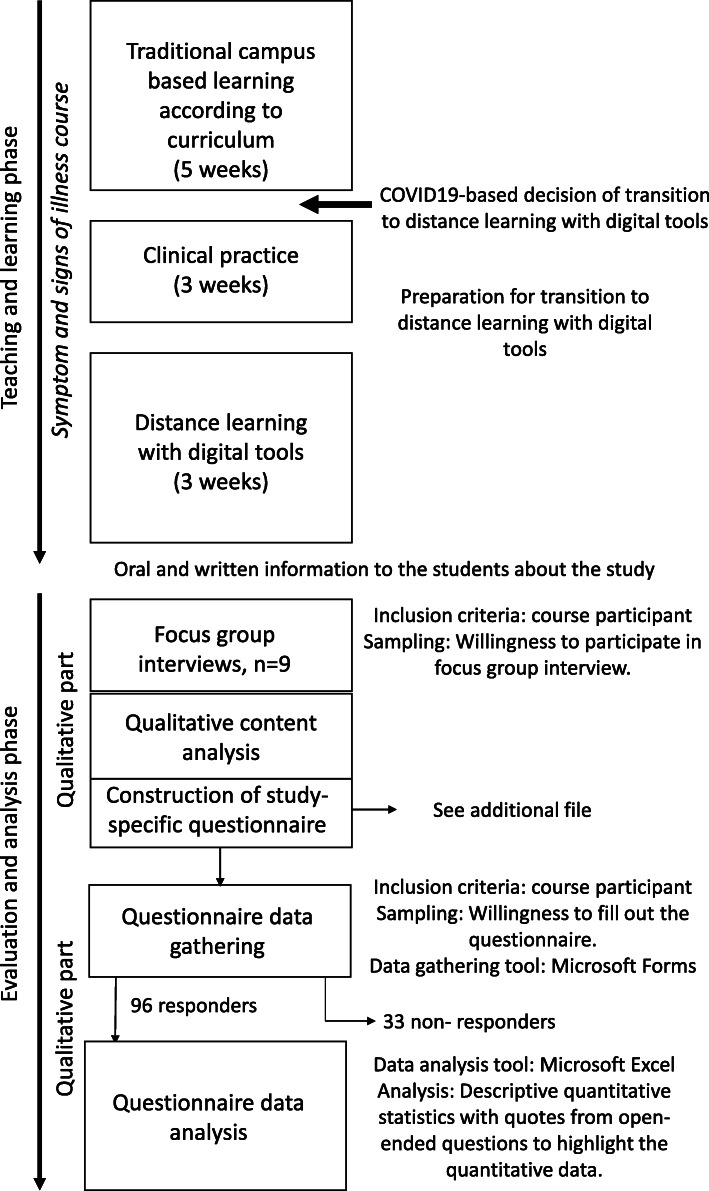
An illustration of the different phases of the study. The study is divided into two phases: The teaching and learning phase and evaluation and analysis phase. Each phase is divided into several steps illustrated by boxes

The overall flow of events in the study and the different evaluation and analysis steps of the study is illustrated in Fig. 1. This research used a combination of qualitative and quantitative methodologies. The evaluation started with focus groups interviews, and the analysis of these interviews formed the basis of the questionnaires used in this study (Additional file 1), which was delivered to all students taking this class. The evaluation was performed immediately after the course ended to avoid recall bias. The research team, consisted of teachers involved in the course, invited students to participate in focus group sessions and a questionnaire after the course through oral (video meeting) and written information (via the learning platform).

### Setting, participants and description of the evaluated course

This study was conducted in Gothenburg, Sweden and included nursing students who participated in the second semester during spring 2020. In general, students participating in nursing program are approximately 90% female and the vast majority are between 20 and 30 years old, these demographics were also observed in present study. The second semester in the Gothenburg University nursing program includes three courses, starting with a 3-week course on microbiology. The second course is an 11-week course called *Symptom and signs of illness* (16.5 ECTS), which focuses on a range of common diagnoses and the pathology, symptoms and signs, treatment, and nursing related to each diagnoses. The semester ends with a 6-week pharmacology course.

When students were in the fifth week of the second course (*Symptom and signs of illness*), the COVID-19 outbreak resulted in the pedagogical transition from traditional campus based lectures to distance learning using digital tools. Originally, this course consisted of several campus based lectures, oral examinations, a written examination, practical training on campus, and 3 weeks of clinical practice. As of the fifth week of this course, students had experienced approximately half of the campus based lectures, two oral examinations, and campus based practical training. Because of the COVID-19 outbreak, the transition from traditional campus based learning to distance learning using digital tools was rapid. The lecturers scheduled for teaching were informed about the change to distance learning using digital tools, but some external lecturers were not familiar with using digital tools as a teaching method. The lecturers had to decide if they had the capability to transition to teaching using digitals tool and prepare the new learning activities within 3 weeks. Some teachers readily adapted to this change, some had more difficulty adapting, and some declined because of an increased clinical burden.

### Learning activities during the distance teaching period

We identified three forms of learning activities during the distance teaching period. The first form was electronic live lectures, which were delivered using Zoom software (San Jose, USA) and included slide shows or white-screen writing by the teacher. The second form was pre-recorded video lectures. These lectures were created using Power Point (Microsoft, Redmond, USA), and included the teacher’s narration of the slide show. Some teachers also included animation highlights to emphasize specific aspects in the slide show. The third form of learning activity was self-study using the course literature and lecture slide shows without direct participation of the teacher. The self-study category was primarily to accommodate the lectures that were canceled by clinicians that had to prioritize clinical work due to the COVID-19 situation.

### Focus group interviews and qualitative analysis

A focus group is a semi-structured group interview in which members interact and exchange their opinions and views on a certain experience in an informal discussion that is focused on a particular topic or issue [12, 13]. The focus group technique is commonly used in nursing education research because of its capacity to generate spontaneous data on multiple perspectives, opinions, and attitudes of participants in a fast and efficient way [13]. Group debriefing should be used after each interview session to verify the initial interpretation and concurrent data analysis [14].

In this study, interactive focus groups were used to capture students’ experiences of the pedagogical transition from traditional campus learning to distance learning using digital tools. All students (*n* = 132) were invited to participate in the focus groups interviews and nine students agreed to participate. Two focus group interviews via video meeting were conducted by the two first authors (UL, KK) for this study to capture what the students had experienced. The focus groups consisted of four students in the first group and five in the second group (Fig. 1) and lasted 60–70 min. The interviews were performed on April 27 and 29, 2020. An interview guide was developed using open-ended questions; for example: “How did you experience the transition from traditional to distance education” and “Have the changes affected your commitment to your studies?” Follow up questions were asked, such as “What has it meant for you?” These questions aimed to elicit deeper narratives where the students reflected on educational aspects and what the pedagogical transition had meant for them. The focus group proceedings were audiotaped and transcribed. In addition, notes were taken during the interviews to capture participants’ non-verbal expressions. The same researchers moderated both groups to ensure consistency of perceptions and analysis of textual and non-textual data.

The transcribed interviews and written observation notes were organized and prepared for qualitative analysis. Data were analyzed using qualitative content analysis following the approach by Graneheim and Lundman [15], as recommended for focus group research [13]. To identify similarities and differences in the student’s experiences, the text was divided into meaning units by identifying sentences containing aspects related to each other through their content and context. The process included identification of codes which were condensed into subthemes and main themes. To ensure the trustworthiness of findings, researchers used member checking and group debriefing, as described by Lincoln and Guba [14]. To confirm the analysis was rigorous, the researchers documented and wrote extensive and detailed notes of the emergent analytical and theoretical insights.

### Questionnaire and quantitative analysis

Data from the focus group interviews were used to construct a web-based questionnaire (Additional file 1). The questionnaire comprised 14 items and included two open-ended questions. The questions were delivered in a semi-scrambled order to avoid revealing the identified dimensions. A Swedish version of the questionnaire was used. All students in the class (*n* = 132) were invited to participate and to answer the questionnaire. A total of 96 students choose to participate (Fig. 1). Data from the questionnaire were collected using Microsoft Forms software (Redmond, USA) during May 12–18, 2020. Quantitative data from the questionnaire were analyzed with descriptive statistics using Microsoft Excel 2016 (Redmond, USA), and presented as percentages or absolute numbers of replies. The students’ comments in the open-ended questions were used to interpret and highlight the quantitative data, and are presented as quotes in the Results section.

### Ethical considerations

This study did not collect sensitive personal information and therefore did not require formal approval from an ethics committee [16]. The Head of Department reviewed and approved this study. An invitation to participate in the focus group interviews and questionnaire was made both orally and in writing to inform potential participants about the study and its purpose. The students were informed that participation in the focus group interviews or taking the questionnaire was viewed as informed consent. Students were informed that study participation was voluntarily and that they could end the interview at any time. The students’ participation in the web-based questionnaire was also voluntary and anonymous.

## Results

### Focus group interviews and qualitative analysis

The focus group interviews provided a spectrum of experiences regarding the pedagogical transition from campus based learning to distance learning using digital tools. The analysis of the focus group interviews through qualitative content analysis resulted in meaning units and were condensed and labelled with 12 codes that were sorted and abstracted into 9 subthemes and, through a process of interpretation, further abstracted in three main themes: *didactic aspects of digital teaching*, *study environment*, and *student’s own resources.*

**Fig. 2:**
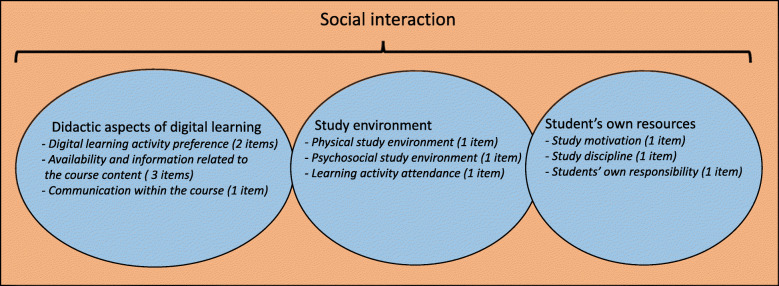
Results from the focus groups interviews regarding the transition to distance teaching using digital tools. The figure visually presents the overall theme, social interaction, and its associations with the three main themes (within circles), which were condensed from the focus group interviews. Social interaction is based on the idea that human development depends more on a person’s interaction with the environment and other individuals than their personal process. The statements in italics in each dimension circle represent the 9 subthemes identified and the12 items covered in the questionnaire. The 2 open-ended questions are not included in the illustration

The first main theme, *didactic aspects of digital learning,* was related to students’ experiences of navigating in digital learning environment in regard to practical and educational aspects of the course. This theme was divided into following subthemes: *digital learning activity preference, and availability and information related to course content* and the *communication within the course*. The second main theme, *study environment,* included subthemes of experiences regarding students’ *physical* and *psychosocial study environment* and *learning activities attendance*. The third main theme, *student’s own resources, reflected* students’ experiences of the subthemes, *study motivation* and *study discipline* and *students own responsibility (*Additional Table 1). Comments for all dimensions were associated with how *social interactions* affected students’ learning process; this emerged as an overall theme for the three dimensions (Fig. 2).

### Questionnaire and quantitative analysis

The questionnaire comprised 14 items based on the codes identified in the focus groups interviews: six items related to *didactic aspects of digital learning*, three items related to *study environment*, and three items covered *students’ own resources*. The questionnaire also included two open-ended questions, one concerning the technical limitations of distance learning using digital tools and one devoted to general comments. These general comments are presented as quotes to illustrate students’ experiences of the pedagogical transition to distance learning using digital tools. The questionnaire was completed by 96 (74%) of the 129 students registered on the course.

### Questionnaire results for didactic aspects of digital learning

**Fig. 3:**
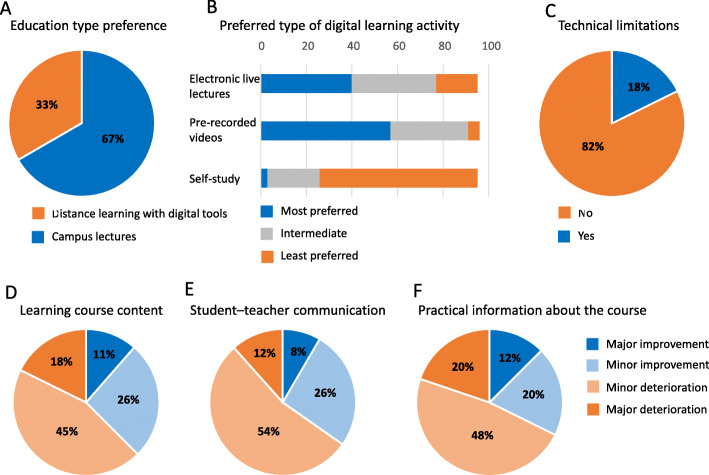
Results relating to the *didactic aspects of digital teaching* dimension. Changes experienced by students after the transition of distance teaching using digital tools in relation to education type preference (**a**), preferred type of digital learning activity (**b**), technical limitations during digital learning activities (**c**), learning course content (**d**), student–teacher communication (**e**), and practical information about the course (**f**). Data are based on 95–96 responses and presented as percentages (**a**, **c**, **d**, **e**, **f**) or absolute numbers of replies (**b**)

Two-thirds of the students reported they preferred regular campus based education to distance learning (Fig. 3a). Three forms of distance learning were used in the course:

*electronic live lectures*, *pre-recorded video lectures*, *and self-study.* Students reported that the most preferred type of learning activity was *pre-recorded video lectures*, followed by *electronic live lectures* (Fig. 3b). However, some students considered *electronic live lectures* the least preferred type of learning activity. Few students reported self-study was the most preferred type of learning activity. *Technical limitations* of distance learning using digital tools were reported by 18% of students (Fig. 3c). The majority of technical limitations were related to the digital tool used for electronic live lectures. Limitations in the teacher’s management of the digital tool as well as Internet limitations were commonly reported.

The didactic aspects dimension also included questions related to *learning course content*, *student–teacher communication*, and *practical information related to the course content.* Students were asked if the pedagogical transition to distance learning using digital tools had impacted their ability to learn the course content. The majority of students reported deterioration in learning course content after the transition to distance learning using digital tools (Fig. 3d). Face-to-face communication with teachers and classmates were experienced as important factors for a deeper understanding of the course content.

*“It has become very clear to me that the human factor and non-verbal communication has an impact to how I learn. It is more difficult to understand and remember the information received though pre-recorded video lectures because I cannot see the person who is talking. To read slides or a book does not give me much if I had not seen or heard the lecture before.”*

*“I think that the collaboration with my classmates did not work so well. Before, I learned through discussions with classmates, asking for help and explanations. I don’t think it is possible to have the same opportunity through digital tools.”*

The pedagogical transition also impacted *student–teacher communication*, with almost two-thirds of the students reporting deterioration in this communication (Fig. 3e).

*“The downside is that you can’t have a personal dialog with the teacher, to be able to go to the teacher and ask questions.”*

A functional deductive approach requires accessible *practical information related to the course*. However, two-thirds of the students experienced deterioration in accessibility to practical information after the transition (Fig. 3f).

*“It has been difficult to know what to learn, when the lectures are held, and how to navigate in the learning platform.”*

### Questionnaire results for study environment

**Fig. 4:**
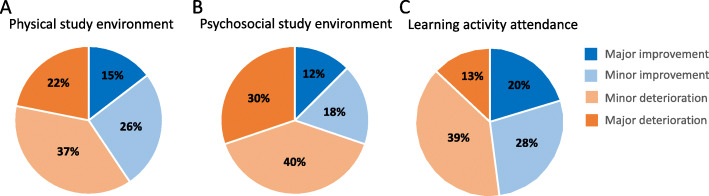
Results relating to the *study environment* dimension during distance teaching using digital tools. Improvements or deteriorations experienced by students after the implementation of distance teaching using digital tools in relation to the physical study environment (**a**), psychosocial study environment (**b**), and attending learning activities (**c**). Data are based on 96 responses and presented as percentages

The second dimension, which included the *physical* and *psychosocial study environment*, was also affected by the pedagogical transition. Students reported deterioration in both the physical and psychosocial study environment (Fig. 4a and b), although the deterioration was more pronounced for the psychosocial study environment. However, other students experienced improvements in both their physical and psychosocial study environment.

*“To not be able to study at the library is also a great loss for me. It provides a very good study environment, with access to course literature and unlimited access to use printers.”*

*“I think you have more time to study when you don’t have to travel back and forth to school.”*

*“I miss my classmates during the breaks and talking to the persons next to me.”*

Despite the deterioration in the study environment, learning activity attendance was not affected by the pedagogical transition (Fig. 4c).

### Questionnaire results for students’ own resources

**Fig. 5:**
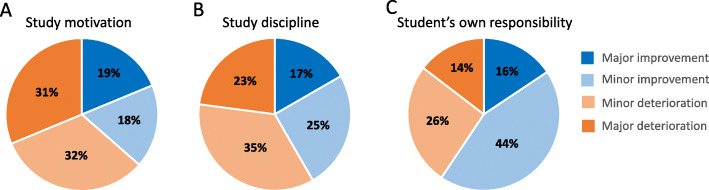
Results relating to the *student’s own resources* dimension during distance teaching using digital tools. Changes experienced by the students after the implementation of distance teaching using digital tools in relation to study motivation (**a**), study discipline (**b**), and students’ own responsibility (**c**). Data are based on 96 responses and presented as percentages

The third dimension was related to the *students’ own resources* and included questions about *study motivation*, *study discipline*, and *students’ own responsibility*. Deterioration was found in study motivation (Fig. 5a). The responses indicated that students felt that face-to-face social interaction with other classmates was important for their study motivation. Some students commented that they lost their social context as students, and compared this to being unemployed:

*“It feels like you are not acquiring knowledge as much as you should, which makes you feel more unsecure and unmotivated.”*

*“When everything is digital, and I don’t have the opportunity to meet my classmates, I don’t have a context. It feels like I am unemployed even though I am not. Much of the motivation disappears since I get my energy and motivation from meeting others.”*

In the absence of study motivation, a well-disciplined student may still meet the course requirements. However, more than half of the students reported a decline in study discipline (Fig. 5b).

*“Distance teaching is not for me since I experience that it is hard to keep track of everything around me and very hard to maintain the discipline when you study at home.”*

The pedagogical transition to distance learning required students to take more responsibility to understand and interpret the course requirements. It also required an increased capability among students to sort and navigate the course information that was communicated via the learning platform. The majority of students reported an increased level of responsibility for their own learning (Fig. 5c).

*“For me, the distance education has contributed to very positive changes within me. Feeling of better control, more responsibility and more freedom to plan everyday life as it suits me. I can do everything in my own pace, which is not very fast.”*

When students meet face-to-face on campus with group projects as a learning activity, they take different roles in the group. The transition to distance learning presented a barrier for students to identify their responsibility in a group setting.

*“The group projects are difficult to manage with digital tools; this puts more responsibility on me as a student, and the question is who takes the initiative in the group.”*

## Discussion

The aim of this study was to describe and evaluate nursing students’ experiences of the pedagogical transition from traditional campus based learning to distance learning using digital tools. The main finding was that the pedagogical transition reduced students’ opportunities for social interactions in their learning process. Even though our results predominantly showed deterioration in all three investigated dimensions, a minority of students favored distance learning using digital tools. Therefore, our results support a blended learning approach, including both campus based and distance learning, which may offer pedagogical benefits, including important social interactions that amplify students’ learning process and motivation.

Similar to other studies [8, 17, 18], this study highlighted the importance of social interactions among students and between students and teachers as an important part of the students’ learning process. The importance of social interaction is not a new concept in teaching. This theory was first developed by Vygotskij [19], and introduced in Europe in 1960 as sociocultural educational theory. According to this theory, human development depends more on a person’s interaction with their environment and other individuals than on a personal process. Vygotskij argued that there is a difference between what a person learns on their own and what that person learns through interaction with others. Depending on the context in which learning occurs, a person can be either at their actual level of development or at their potential level of development. The actual level of development refers to when a person learns something by their own power, and the potential level of development is when learning opportunities increase through interactions with others with more knowledge and experience [19, 20]. The need for social interaction in distance education is evident, and poses a major challenge for teachers to create learning activities that support social interaction. For example, digital group assignments, digital group discussions, or group chats may be good ways to increase students’ social interaction in the digital environment and enable students to reach their potential level of development.

The first dimension of this study was *didactic aspects in distance learning using digital tools*. Digital tools in education are important because they may facilitate learning and also because they change how we learn [21]. Säljö [21] argued that fitting digital tools into an established way of teaching may create suboptimal learning conditions. In the present study, students were not always satisfied with the digital learning activities and reported difficulty finding and understanding the information that was communicated through the digital learning platform. Therefore, the results of this study emphasized the importance of adapted learning activities, clear instructions, and a visible course structure when using digital tools. These results are in line with Delgaty [22], who discussed the importance of clear guidance surrounding strategies to support changes in distance learning. The rapid transition in the context of the COVID-19 pandemic forced educators to incorporate digital tools in a course planed for traditional campus based teaching. A longer preparation time for the pedagogical transition to digital tools might have resulted in more positive experiences of distance learning. It is the teacher’s responsibility to include a clear structure when designing learning activities that does not leave students on their own in their learning process [17, 23]. This statement was echoed in a recent publication by Porter et al. [24] that described the academic experiences of transitioning to blended online learning, and highlighted the importance of teachers’ preparation and planning in the pedagogical transition to distance learning using digital tools. Adapting learning activities to a digital environment and improving practical information about the course and the course structure are key components in creating successful distance learning.

Previous research showed that there were differences between the digital learning activities that students preferred and what leads to effective and efficient learning [25]. In this study, students preferred pre-recorded video lectures to electronic live lectures or study on their own. The preference for electronic live lectures might have been reduced because of technical problems with this form of learning activity. The students reported that the major benefits with pre-recorded video lectures were that they could be completed at any time, at their own pace, and viewed multiple times. However, it remains unclear whether students learn best through pre-recorded video lectures; further studies are needed to answer such questions.

In terms of the second dimension, participating students stated that the lack of social interaction with other students and teachers negatively affected their psychosocial study environment. Consistent with this finding, Walsh [18] showed that reduced social interaction had a negative effect on nursing students’ mental health. In a university environment, emphasis is placed on individual achievement, but students clearly stated the importance of social processes in how they coped with the demands of higher education [18]. Our findings and previous research support the idea that teachers need to develop didactic strategies that compensate for the lack of social interaction to improve the psychosocial study environment in distance learning. This is especially important as educators have limited potential to influence the physical study environment during distance teaching.

Finally, in the third dimension, students reported their own responsibility for their studies had increased as a positive effect of the pedagogical transition. Transition from campus based learning to distance learning placed more responsibility on students’ capacity to adapt to changes associated with this transition. Salmon [23] claimed that students need to develop new skills to manage information and knowledge obtained in a digital learning environment. We suggest that teachers may need to take a more active role in improving digital literacy to reduce the number of students struggling with the technical aspects of the digital tools. If students have difficulty adapting to these changes and the new study environment in addition to academic requirements, it may result in decreased motivation and high occurrences of issues such as anxiety, dissatisfaction, stress and social isolation [26]. In addition, Nilsson et al. [27] highlighted the need for educational organizations to maintain students’ motivation, which may be more important in a digital setting than a traditional setting. Further, a decrease in social interaction may lead to social isolation, which may also affect students’ motivation. Students need to maintain their motivation without the constant interaction that campus based learning offers, and must be disciplined to meet the course requirements, although study discipline may be difficult for teachers to influence. By creating tasks and assignments that stimulate students to interact with each other or with teachers, students’ motivation may be improved and social isolation prevented [17]. Blended teaching methods have been suggested as an effective method to reach students with low motivation [28]. This also accords with previous research by Jowsey et al. [29] which suggests that when blended learning is delivered purposefully and effectively in terms of managing and supporting student active learning, it positively influences the achievements of students.

### Limitations and strengths

This study was limited because it was only performed during a single course and at one university. It was also limited in that the pedagogical transition to distance learning was not pre-planned, and therefore might not have been optimally designed. Few participants in the focus groups and lack of validated questionnaires addressing this research topic are also limitations. Our questionnaire was based on the focus groups interviews responses and had not undergone any validation processes. Hence, the reliability and validity are therefore unknown. The major strengths of this study were the high participation rate (74%) and that the same students experienced the two forms of teaching within a single course.

## Conclusion

The successful implementation of digital tools depends on several factors, such as students’ level of motivation and level of social interaction between students and teachers. It is clear that there is variability in students’ preferences for education forms and digital learning activities, which have an impact on how teachers design courses. Therefore, a blended learning structure with both campus based and distance learning using digital tools should be considered, with a focus on the components of the course that are best suited for distance learning. These considerations need to reflect the topics under study as well as the specific learning objectives of the course. The lack of social interaction in distance learning is a major challenge, and teachers need to create learning activities that improve social interactions. Distance learning using digital tools also requires a well-designed course structure to compensate for the lack of social interaction.

## Supplementary Information

**Additional file 1.** Translation of the study-specific web-based questionnaire used in the study. A Swedish version of the questionnaire was administered to participating students.

**Additional file 2: Table 1**. The Analytical process for the main theme *Didactic aspects of digital teaching.*

## Data Availability

The datasets used and analyzed during the present study are available from the corresponding author on reasonable request.

## Abbreviation

ECTS: European Credit Transfer System
