# Supplementary material for: Nursing students’ experiences of a pedagogical transition from campus learning to distance learning using digital tools
Source: BMC Nurs. 2021 Jan 19;20:23. doi: 10.1186/s12912-021-00542-1 (PMC7814979; doi:10.1186/s12912-021-00542-1)
Supplement: Supplementary file 1 — Additional file 1. Translation of the study-specific web-based questionnaire used in the study. A Swedish version of the questionnaire was administered to participating students. [file 12912_2021_542_MOESM1_ESM.docx]

# Nursing students’ experiences of a transition from campus learning to distance learning using digital tools

Authors: Ulrica Langegård^1^*, Kiana Kiani^1^*, Susanne Nielsen^2^, Per-Arne Svensson^1,2^

Affiliations: ^1^Institute of Health and Care Sciences, Sahlgrenska Academy at University of Gothenburg, Gothenburg, Sweden; ^2^Department of Molecular and Clinical Medicine, Institute of Medicine, Sahlgrenska Academy at University of Gothenburg, Gothenburg, Sweden

-------------------------------------------------------------------------------------------------------------

**Additional file 1.** Translation of the study-specific web-based questionnaire used in the study. A Swedish version of the questionnaire was administered to participating students.

----------------------------------------------------------------------------------------------------------------------------------

**An evaluation of students’ experiences of the transition to distance teaching using digital tools during the COVID-19 situation**

The COVID-19 pandemic during the spring of 2020 led to major changes in the teaching format of the *Symptom and signs* course (OM6210). For you, this has meant that the second half of the OM6210 course was changed to distance teaching using digital tools. We want to evaluate the transition to distance learning using digital tools.
This evaluation will support the development of teaching at the department, and may also be published as scientific work. By answering the questionnaire, you are consenting to your answers being included in data that may be published. The questionnaire is anonymous. When answering the questions, we want you to assess the teaching methods used, not the ability of individual teachers, materials, or subject areas that specifically interest you.

Please make a joint assessment of all lectures that used this format.

1. Which form of teaching do you prefer from the following two options?

-Campus lectures

-Distance learning using digital tools

2. We have identified that distance teaching with digital tools was essentially conducted in three different ways: 1) electronic live lectures (via zoom); 2) pre-recorded video lectures, including the teachers narration of a slide show; and 3) self-study using the course literature and lecture slide shows.

Please rank from 1–3, where 1 is the lecture form that you preferred most and 3 is the lecture form that you preferred least.

-Electronic live lectures

-Pre-recorded video lectures

-Self-study using course literature

In the following questions, we want you to put a cross in the parentheses that best applies to you.

3. How has the transition to distance teaching using digital tools affected your own responsibility for your studies?

-Major improvement

-Minor improvement

-Minor deterioration

-Major deterioration

4. How has the transition to distance teaching using digital tools affected the motivation for your studies?

-Major improvement

-Minor improvement

-Minor deterioration

-Major deterioration

5. How has the transition to distance learning using digital tools affected your ability to learn the content of the course?

-Major improvement

-Minor improvement

-Minor deterioration

-Major deterioration

6. How has the transition to distance teaching using digital tools affected your physical study environment?

-Major improvement

-Minor improvement

-Minor deterioration

-Major deterioration

7. How has the transition to distance teaching using digital tools affected your psychosocial study environment?

-Major improvement

-Minor improvement

-Minor deterioration

-Major deterioration

8. How has the transition to distance teaching using digital tools changed the availability of practical information about the course?

-Major improvement

-Minor improvement

-Minor deterioration

-Major deterioration

9. How has the transition to distance teaching using digital tools affected your communication with your teachers in this course?

-Major improvement

-Minor improvement

-Minor deterioration

-Major deterioration

10. How has the transition to distance teaching using digital tools affected your study discipline?

-Major improvement

-Minor improvement

-Minor deterioration

-Major deterioration

11. How has the transition to distance teaching using digital tools affected your attendance of learning activities in this course?

-Major improvement

-Minor improvement

-Minor deterioration

-Major deterioration

12. Has the transition to distance teaching using digital tools meant any technical limitations?

-Yes

-No

13. If you answered yes to question 12, what kind of technical limitations did you experience during the transition to distance teaching using digital tools?

[open question]

14. Is there anything else you want to share with us in relation to the transition to distance teaching using digital tools?

[open question]
