# Supplementary material for: Nursing students’ experiences of a pedagogical transition from campus learning to distance learning using digital tools
Source: BMC Nurs. 2021 Jan 19;20:23. doi: 10.1186/s12912-021-00542-1 (PMC7814979; doi:10.1186/s12912-021-00542-1)
Supplement: Supplementary file 2 — Additional file 2: Table 1. The Analytical process for the main theme Didactic aspects of digital teaching. [file 12912_2021_542_MOESM2_ESM.docx]

| Additional Table 1 | The Analytical process for the main theme *Didactic aspects of digital teaching* | | |
| --- | --- | --- | --- |
| Condensed meaning unit  Description close to the text | Condensed meaning unit /  Interpretation of the underlying meaning/ Code | Subtheme | Main theme |
| The pre-recorded video lecture with slide show makes the lecture more available and it can be played back and forward to better follow the content of the lecture | Preference for the pre-recorded video lecture | *Digital learning activity* *preference* | *Didactic aspects of digital learning* |
| With only the slide shows to follow it is sometimes difficult to study on your own | Less preference for lecture by only slide shows |  |  |
| The digital learning platform is not always made for digital use only which makes it less user friendly | The learning platforms limitations in digital settings | *Availability and information related to course content* |  |
| Difficulty to follow the changes in curriculum because of too much information at once | Large volume of information during the pedagogical transition |  |  |
| Confusing information through the digital learning platform | Unclear and unorganized information through learning platform |  |  |
| A decrease in personal communication with teachers and lecturer about the content of the course or lecture as a mean to clarify things. | Less opportunity to have face to face communication with teachers or lecturer | *The* *communication within the course* |  |
|  | The Analytical process for the main theme *Study environment* | | |
| Condensed meaning unit  Description close to the text | Condensed meaning unit /  Interpretation of the underlying meaning/ Code | Subtheme | Main theme |
| Easier to study at home where students could be more focused and had access to all the course litterateur while listening to an electronic live lecture or going through slide shows | More focused and more access to course literature | *Physical* *study environment* | *Study environment* |
| Missing classmates in similar situation for discussions and as a way to boost the study motivation | The social interaction with other student in similar situation is important | *Psychosocial study environment* |  |
| More participation in an electronic live lecture than a campus-based lecture. | More availability and attendance through digital settings | *Learning activities attendance* |  |
|  | The Analytical process for the main theme *Student’s own resources* | | |
| Condensed meaning unit  Description close to the text | Condensed meaning unit /  Interpretation of the underlying meaning/ Code | Subtheme | Main theme |
| Difficulty to stay motivated at home because of interference of external factors | External factors´ effect on study motivation | *Study motivation* | *Student’s own resources* |
| Take more active part in learning about the objectives or the requirements in the course and have more of a greater will to achieve these | More willingness and discipline to achieve course requirements | *Study discipline* |  |
| Take more personal responsibility following the requirements in the course and rely less on other classmates | More personal responsibility | *Students own responsibility* |  |
| The table describes the analysis process through meaning codes, sub themes and the main themes of the qualitative content analysis | | | |
